# Editorial: Exploring the oral-gut microbiome interactions: pathways to therapeutic strategies and implications for systemic health

**DOI:** 10.3389/fmicb.2025.1691238

**Published:** 2025-10-03

**Authors:** Romain Lan, Lucas De Paula Ramos, Zhangran Chen, Florence Carrouel

**Affiliations:** ^1^Laboratory “Health Systemic Process” (P2S), UR4129, University Claude Bernard Lyon 1, University of Lyon, Lyon, France; ^2^Laboratory ADES, Aix Marseille University, CNRS, EFS, Marseille, France; ^3^School of Dentistry, Federal University of Alfenas—UNIFAL, Alfenas, Brazil; ^4^Shenzhen Wedge Microbiology Research Co., Ltd., Shenzhen, China

**Keywords:** oral–gut axis, microbiome methodology, microbial ecology, systemic health, prevention and public health

The field of microbiome research has rapidly expanded in recent years, with particular attention paid to the interplay between the oral and gut ecosystems (Kunath et al., 2024). Traditionally investigated as distinct entities, these microbial communities are now recognized as interconnected through what is increasingly referred to as the oral–gut axis (Tanwar et al., 2024; Xu et al., 2025). The contributions gathered in this Research Topic provide new insights into methodological challenges, ecological mechanisms, clinical implications, and translational opportunities for prevention and public health. Together, they highlight both the promise and the complexity of integrating oral and gut microbiome science into a broader framework of systemic health.

One of the strongest messages emerging concerns methodology. Long-read sequencing with PacBio HiFi has been shown to optimize reference databases for both oral and gut datasets, substantially improving species-level taxonomic assignment and biomarker discovery compared with classical resources (Han et al.). By integrating PacBio-derived amplicon sequence variants into existing oral databases, coverage is enhanced without inflating database size, offering a practical way forward for standardization. Equally, the way samples are collected and stored has significant impact: lyophilized stool skews the Firmicutes: Bacteroidetes ratio and amplifies archaeal signals, while fresh saliva preserves the greatest diversity and thawed dental supernatant captures more richness than pellet or composite fractions (Superdock et al.). Such findings underline that methodological details—whether in pre-analytical handling or in taxonomic assignment—can drive differences as large as those attributed to biology itself (Superdock et al.; Han et al.). Without harmonized pipelines, even preventive interventions such as dietary or oral hygiene strategies tested in one context may not translate reliably to another. Methodological rigor is not only a technical concern but also a prerequisite for embedding microbiome science into effective prevention strategies against chronic diseases (Bourgeois et al., 2025).

Methodology is crucial because the oral–gut axis is not merely conceptual; it reflects real ecological connectivity. In healthy subjects, dozens of amplicon sequence variants are consistently shared between the oral cavity and gut, with the majority more abundant in the mouth, suggesting a steady oral-to-gut transfer across the lifespan (Costa et al.). After gastrectomy, increased gut microbial evenness and the emergence of oral taxa such as *Rothia* and *Lactobacillus* in the intestine imply that alteration of gastric barriers facilitates microbial redistribution (Komori et al.). In alcohol dependence, oral contributions to gut communities are significantly greater than in controls, with SourceTracker attributing more than 5% of gut composition to oral origin and network analysis highlighting *Prevotella* and *Neisseria* as potential driver taxa (Hu et al.). By contrast, in a cohort of elders with well-maintained oral conditions and more than 26 natural teeth, a minimal oral–gut overlap was reported, with low shared-amplicon sequence variant ratios and stable gut profiles despite a distinct oral metabolome (Nishimoto et al.). This suggests that barrier integrity and dental care can constrain transmission along the axis.

The clinical scope of the oral–gut axis is equally broad. In children, extrinsic black stain is paradoxically associated with reduced caries, and distinct bacterial signatures are detectable both in plaque and feces, with protective taxa enriched in the stain group and pathogenic genera predominating in caries-prone groups (Zheng et al.). In adults with autism spectrum disorder, a strikingly high prevalence of periodontitis is associated with frequent detection of red-complex bacteria and with digestive comorbidities and medication use, thereby linking oral dysbiosis to systemic vulnerabilities in a neglected population (Berbé et al.). Emerging causal evidence further supports this axis. Data have suggested associations between specific oral taxa and colorectal cancer risk. *Haemophilus* and *Fusobacterium* were indicated as potential risk factors, whereas *Metamycoplasma salivarium* and *Mogibacterium pumilum* appeared protective (Zhang et al.).

Beyond these specific associations, several contributions highlight chronic diseases where the oral–gut axis may act as a hidden mediator. Rheumatoid arthritis research continues to underscore the role of *Porphyromonas gingivalis* in protein citrullination, while bibliometric mapping reveals an increasing focus on how oral and gut ecosystems together shape autoimmune susceptibility (Lin et al.). A complementary bibliometric synthesis of studies involving the *Muribaculaceae* (S24-7) illustrates how shared microbial families connect oral and gut studies through short-chain fatty acids production and carbohydrate metabolism (Gao et al.). These observations emphasize that the oral–gut axis represents just one example of a broader network of microbiome-mediated connections across organ systems. In particular, respiratory pathways are increasingly explored through the lens of oral–respiratory and gut–lung axes, underscoring how microbial cross-talk extends beyond digestion to immune and inflammatory regulation.

The scope also extends to respiratory and ear, nose, and throat conditions. In laryngeal cough, patients display reduced microbial diversity and distinct profiles involving genera such as *Veillonella* and *Streptococcus*; treatment with a traditional herbal formula is associated with both clinical improvement and microbial shifts (Yu et al.). In asthma, the gut–lung axis is increasingly understood through the lens of microbial metabolites, with short-chain fatty acids, bile acids, and amino acid pathways acting as modulators of inflammation; here again, traditional medicine approaches are highlighted as potential tools for influencing these microbial–metabolite interactions (Lu et al.). Tongue-coating profiling in Parkinson's disease provides further evidence for the oral–gut–brain axis. Integrated microbiome–metabolome analysis revealed microbial and metabolic alterations, supporting tongue coating as a potential non-invasive diagnostic approach to systemic neurodegeneration (Yang et al.).

Diet and metabolites emerge as recurring themes. The identification of short-chain fatty acids producers in the *Muribaculaceae* review (Gao et al.) and in asthma (Lu et al.) studies supports the notion that fiber-rich diets and targeted nutritional interventions can modulate both oral and gut communities at the same time (Lin et al.). These pathways underscore diet as a preventive lever operating across multiple body sites. Oral hygiene similarly surfaces as a systemic preventive measure: maintaining a balanced oral community may limit the seeding of pathobionts to the gut and mitigate risks extending far beyond the mouth. Evidence from studies on shared taxa, on the caries–black stain paradox, and on vulnerable groups such as those with autism suggest that oral care should be embedded in systemic health strategies (Zheng et al.; Berbé et al.). Findings in pregnant populations also underline that simple habits such as twice-daily toothbrushing reduce the load of key pathogens and interdental bleeding, supporting early prophylactic guidance during pregnancy (Carrouel et al., 2023).

Thus, oral and gut microbiomes are not separate entities but nodes in a connected ecological corridor. Microbes migrate along this axis under normal conditions, and such migration intensifies when barriers are breached or when systemic diseases alter the host environment (Maki et al., 2021). Methodological rigor is essential to discern true biological signals from artifacts. Dietary habits and oral hygiene are not only local factors but also systemic levers influencing outcomes in fields as diverse as rheumatology, nephrology, gastroenterology, psychiatry, and pulmonology (Botelho et al., 2022).

At the same time, important gaps remain. Most associations described are correlational; causality requires interventional studies that track strains across sites, manipulate diets or oral hygiene regimens, and monitor systemic outcomes (Shoer et al., 2023). Mechanistic pathways involving short-chain fatty acids, bile acids, and microbial translocation need to be confirmed in controlled settings. Consensus on standardized storage, sequencing, and analysis pipelines is needed to enable robust multicenter trials (Roume et al., 2023; Metwaly et al., 2025).

The oral–gut axis is moving from hypothesis to framework with direct implications for clinical practice and health policy. For researchers, the next step is to integrate standardized methods with strain-resolved, multi-omic analyses capable of testing causality. For clinicians and policymakers, the immediate opportunity lies in embedding oral hygiene promotion and dietary guidance into chronic disease prevention strategies. By acting simultaneously on the mouth and the gut, microbiome science can be harnessed not only to understand disease mechanisms but also to design pragmatic, prevention-oriented interventions (Zhu et al., 2025).

Overall, the contributions assembled in this Research Topic—spanning methodological innovation, mechanistic exploration, clinical associations, and preventive perspectives—make a compelling case that the oral–gut axis should be a central theme in the future of microbiome research and public health.
